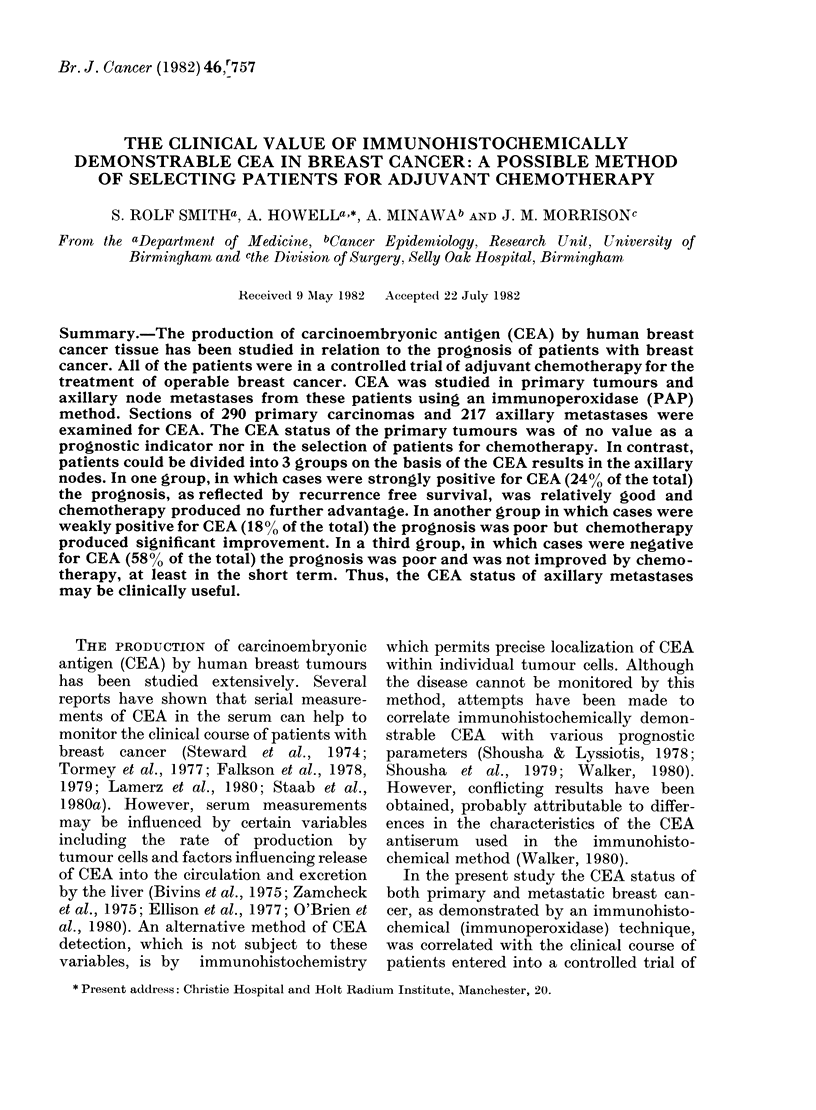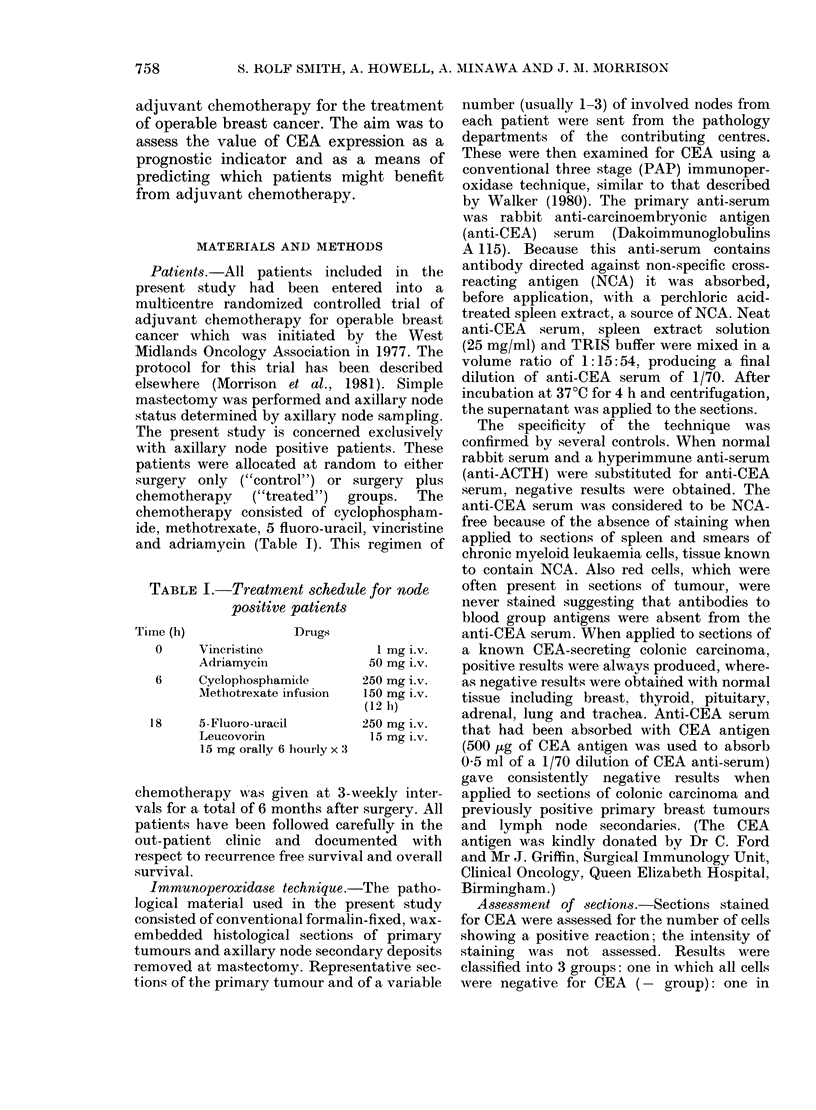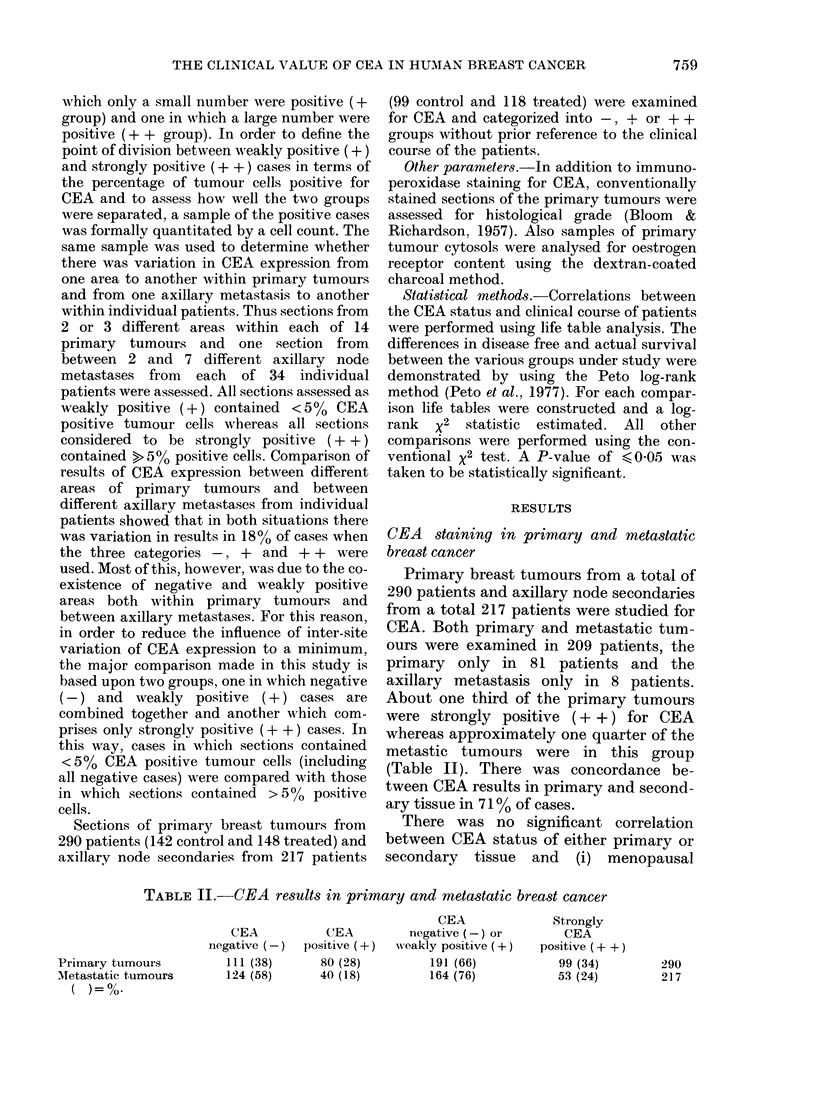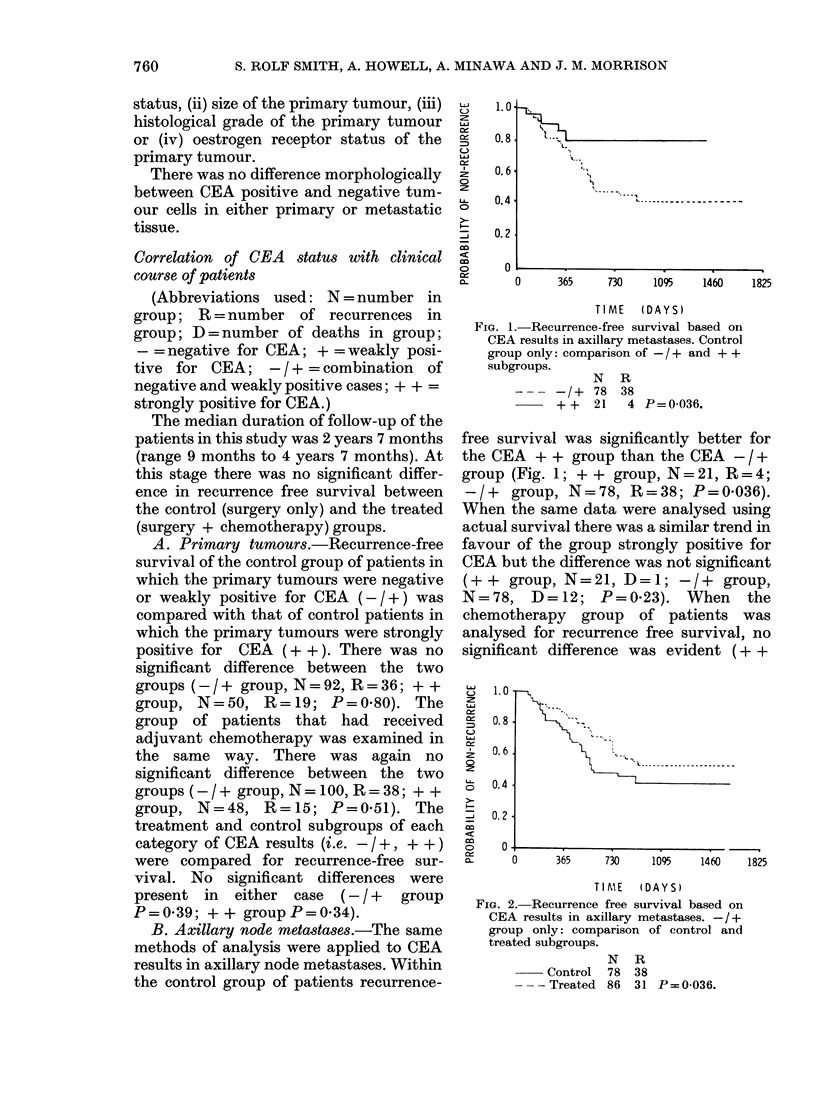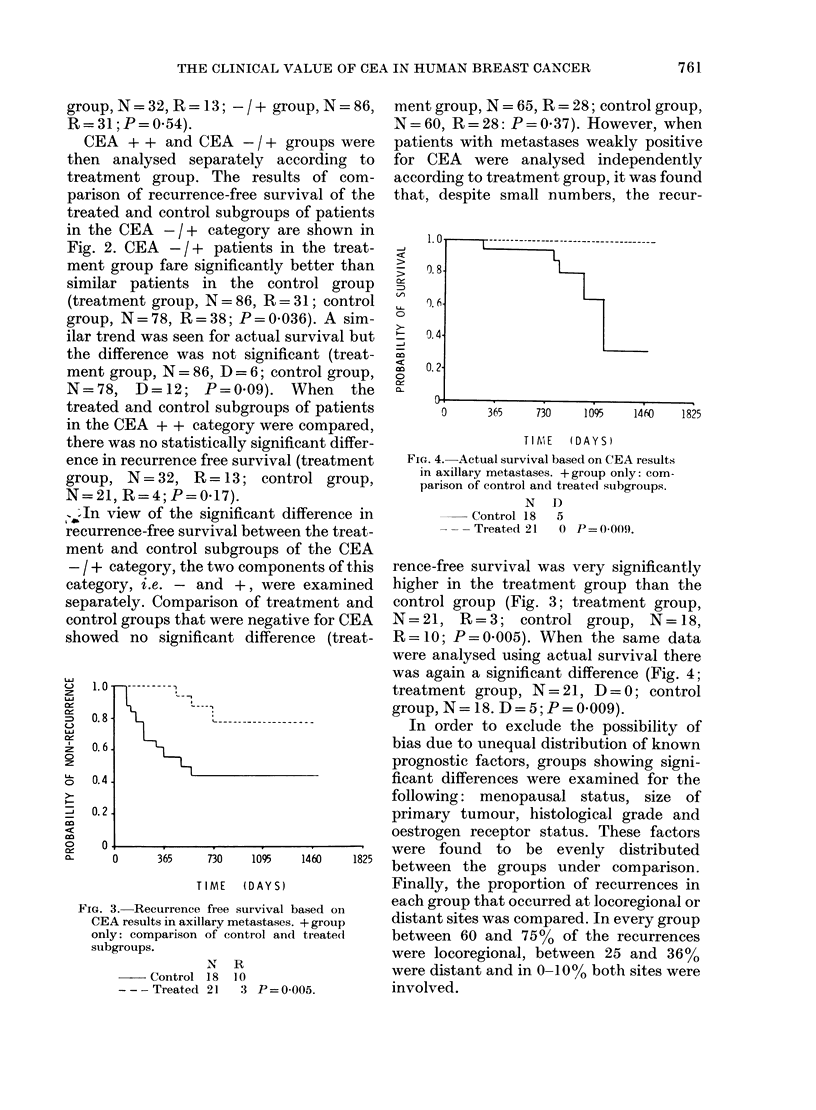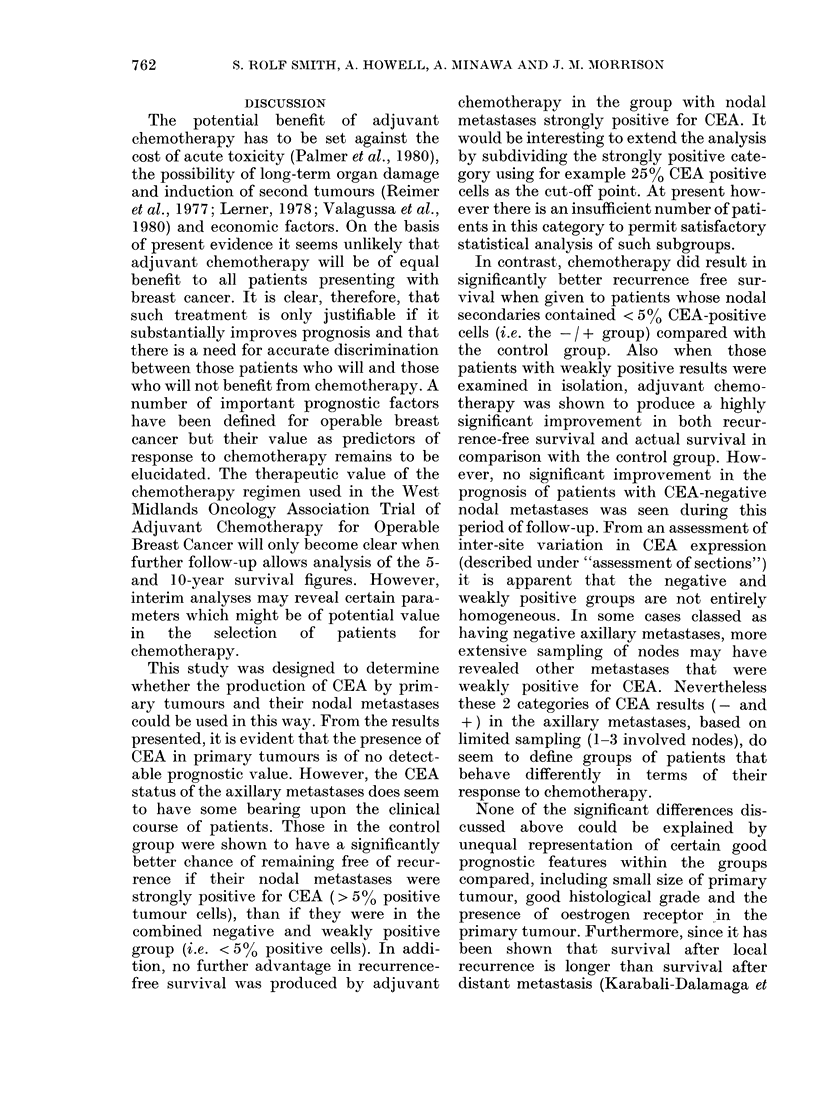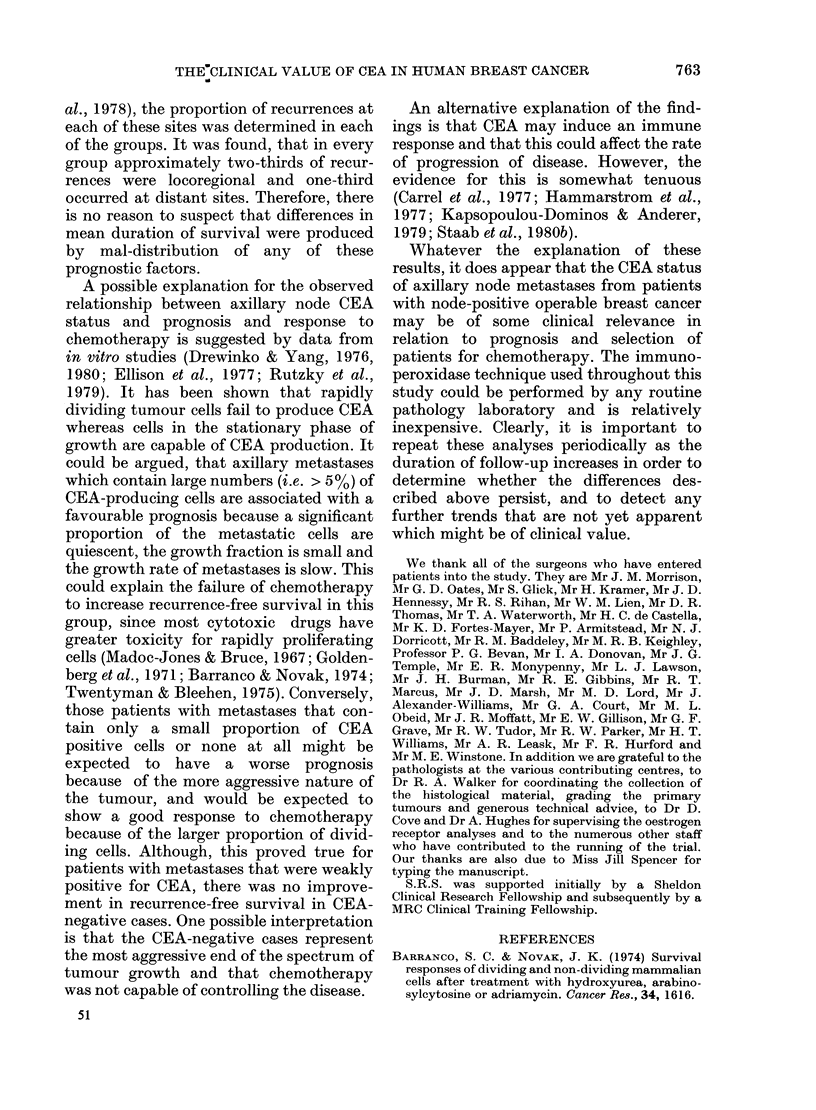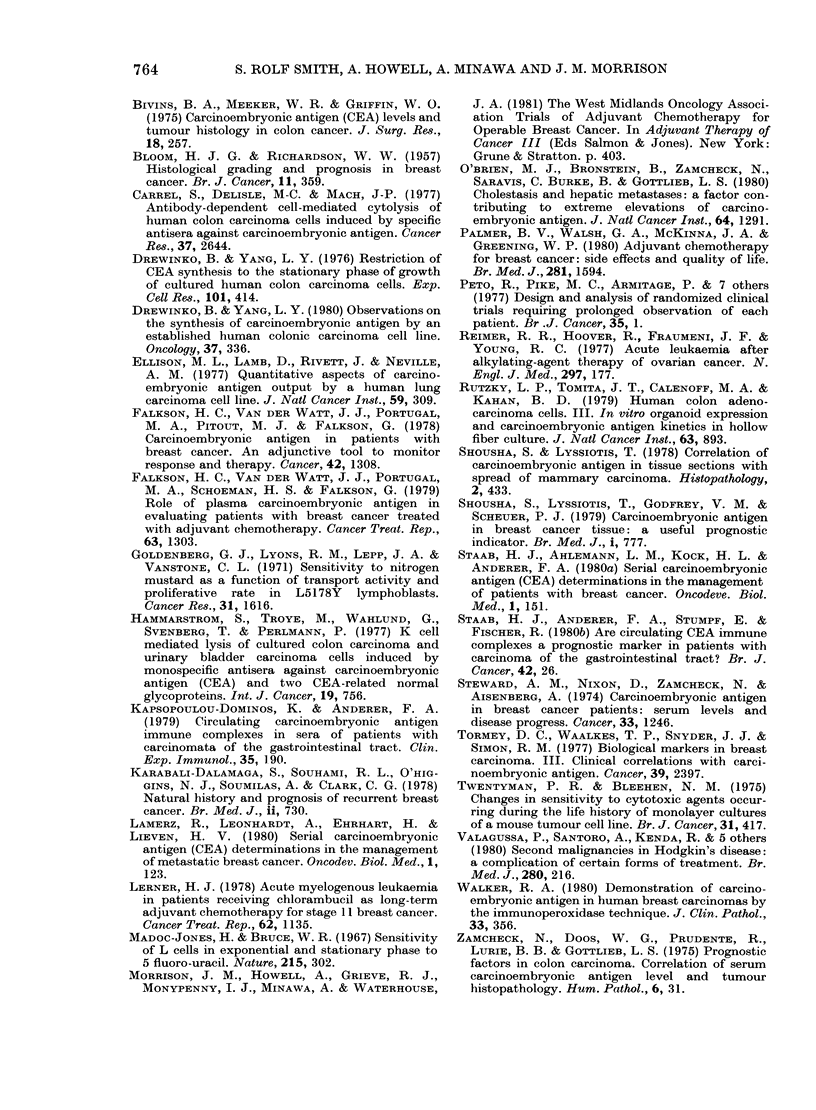# The clinical value of immunohistochemically demonstrable CEA in breast cancer: a possible method of selecting patients for adjuvant chemotherapy.

**DOI:** 10.1038/bjc.1982.268

**Published:** 1982-11

**Authors:** S. R. Smith, A. Howell, A. Minawa, J. M. Morrison

## Abstract

The production of carcinoembryonic antigen (CEA) by human breast cancer tissue has been studied in relation to the prognosis of patients with breast cancer. All of the patients were in a controlled trial of adjuvant chemotherapy for the treatment of operable breast cancer. CEA was studied in primary tumours and axillary node metastases from these patients using an immunoperoxidase (PAP) method. Sections of 290 primary carcinomas and 217 axillary metastases were examined for CEA. The CEA status of the primary tumours was of no value as a prognostic indicator nor in the selection of patients for chemotherapy. In contrast, patients could be divided into 3 groups on the basis of the CEA results in the axillary nodes. In one group, in which cases were strongly positive for CEA (24% of the total) the prognosis, as reflected by recurrence free survival, was relatively good and chemotherapy produced no further advantage. In another group in which cases were weakly positive for CEA (18% of the total) the prognosis was poor but chemotherapy produced significant improvement. In a third group, in which cases were negative for CEA (58% of the total) the prognosis was poor and was not improved by chemotherapy, at least in the short term. Thus, the CEA status of axillary metastases may be clinically useful.


Br. J. Cancer (1982) 46, 757

### THE CLINICAL VALUE OF IMMUNOHISTOCHEMICALLY DEMONSTRABLE CEA IN BREAST CANCER: A POSSIBLE METHOD OF SELECTING PATIENTS FOR ADJUVANT CHEMOTHERAPY

S. ROLF SMITH[a], A. HOWELL[a,*], A. MINAWA[b] AND J. M. MORRISON[c]

*From the [a]Department of Medicine, [b]Cancer Epidemiology, Research Unit, University of Birmingham and [c]the Division of Surgery, Selly Oak Hospital, Birmingham*

Received 9 May 1982 Accepted 22 July 1982

**Summary.—The production of carcinoembryonic antigen (CEA) by human breast cancer tissue has been studied in relation to the prognosis of patients with breast cancer. All of the patients were in a controlled trial of adjuvant chemotherapy for the treatment of operable breast cancer. CEA was studied in primary tumours and axillary node metastases from these patients using an immunoperoxidase (PAP) method. Sections of 290 primary carcinomas and 217 axillary metastases were examined for CEA. The CEA status of the primary tumours was of no value as a prognostic indicator nor in the selection of patients for chemotherapy. In contrast, patients could be divided into 3 groups on the basis of the CEA results in the axillary nodes. In one group, in which cases were strongly positive for CEA (24% of the total) the prognosis, as reflected by recurrence free survival, was relatively good and chemotherapy produced no further advantage. In another group in which cases were weakly positive for CEA (18% of the total) the prognosis was poor but chemotherapy produced significant improvement. In a third group, in which cases were negative for CEA (58% of the total) the prognosis was poor and was not improved by chemotherapy, at least in the short term. Thus, the CEA status of axillary metastases may be clinically useful.**

THE PRODUCTION of carcinoembryonic antigen (CEA) by human breast tumours has been studied extensively. Several reports have shown that serial measurements of CEA in the serum can help to monitor the clinical course of patients with breast cancer (Steward *et al.*, 1974; Tormey *et al.*, 1977; Falkson *et al.*, 1978, 1979; Lamerz *et al.*, 1980; Staab *et al.*, 1980*a*). However, serum measurements may be influenced by certain variables including the rate of production by tumour cells and factors influencing release of CEA into the circulation and excretion by the liver (Bivins *et al.*, 1975; Zamcheck *et al.*, 1975; Ellison *et al.*, 1977; O'Brien *et al.*, 1980). An alternative method of CEA detection, which is not subject to these variables, is by immunohistochemistry which permits precise localization of CEA within individual tumour cells. Although the disease cannot be monitored by this method, attempts have been made to correlate immunohistochemically demonstrable CEA with various prognostic parameters (Shousha & Lyssiotis, 1978; Shousha *et al.*, 1979; Walker, 1980). However, conflicting results have been obtained, probably attributable to differences in the characteristics of the CEA antiserum used in the immunohistochemical method (Walker, 1980).

In the present study the CEA status of both primary and metastatic breast cancer, as demonstrated by an immunohistochemical (immunoperoxidase) technique, was correlated with the clinical course of patients entered into a controlled trial of

* Present address: Christie Hospital and Holt Radium Institute, Manchester, 20.

adjuvant chemotherapy for the treatment of operable breast cancer. The aim was to assess the value of CEA expression as a prognostic indicator and as a means of predicting which patients might benefit from adjuvant chemotherapy.

### MATERIALS AND METHODS

*Patients.*—All patients included in the present study had been entered into a multicentre randomized controlled trial of adjuvant chemotherapy for operable breast cancer which was initiated by the West Midlands Oncology Association in 1977. The protocol for this trial has been described elsewhere (Morrison *et al.*, 1981). Simple mastectomy was performed and axillary node status determined by axillary node sampling. The present study is concerned exclusively with axillary node positive patients. These patients were allocated at random to either surgery only ("control") or surgery plus chemotherapy ("treated") groups. The chemotherapy consisted of cyclophosphamide, methotrexate, 5 fluoro-uracil, vincristine and adriamycin (Table I). This regimen of

TABLE I.—*Treatment schedule for node positive patients*

| Time (h) | Drugs | |
|---|---|---|
| 0 | Vincristine | 1 mg i.v. |
| | Adriamycin | 50 mg i.v. |
| 6 | Cyclophosphamide | 250 mg i.v. |
| | Methotrexate infusion | 150 mg i.v. (12 h) |
| 18 | 5-Fluoro-uracil | 250 mg i.v. |
| | Leucovorin | 15 mg i.v. |
| | 15 mg orally 6 hourly × 3 | |

chemotherapy was given at 3-weekly intervals for a total of 6 months after surgery. All patients have been followed carefully in the out-patient clinic and documented with respect to recurrence free survival and overall survival.

*Immunoperoxidase technique.*—The pathological material used in the present study consisted of conventional formalin-fixed, wax-embedded histological sections of primary tumours and axillary node secondary deposits removed at mastectomy. Representative sections of the primary tumour and of a variable number (usually 1–3) of involved nodes from each patient were sent from the pathology departments of the contributing centres. These were then examined for CEA using a conventional three stage (PAP) immunoperoxidase technique, similar to that described by Walker (1980). The primary anti-serum was rabbit anti-carcinoembryonic antigen (anti-CEA) serum (Dakoimmunoglobulins A 115). Because this anti-serum contains antibody directed against non-specific cross-reacting antigen (NCA) it was absorbed, before application, with a perchloric acid-treated spleen extract, a source of NCA. Neat anti-CEA serum, spleen extract solution (25 mg/ml) and TRIS buffer were mixed in a volume ratio of 1:15:54, producing a final dilution of anti-CEA serum of 1/70. After incubation at 37°C for 4 h and centrifugation, the supernatant was applied to the sections.

The specificity of the technique was confirmed by several controls. When normal rabbit serum and a hyperimmune anti-serum (anti-ACTH) were substituted for anti-CEA serum, negative results were obtained. The anti-CEA serum was considered to be NCA-free because of the absence of staining when applied to sections of spleen and smears of chronic myeloid leukaemia cells, tissue known to contain NCA. Also red cells, which were often present in sections of tumour, were never stained suggesting that antibodies to blood group antigens were absent from the anti-CEA serum. When applied to sections of a known CEA-secreting colonic carcinoma, positive results were always produced, whereas negative results were obtained with normal tissue including breast, thyroid, pituitary, adrenal, lung and trachea. Anti-CEA serum that had been absorbed with CEA antigen (500 $\mu$g of CEA antigen was used to absorb 0·5 ml of a 1/70 dilution of CEA anti-serum) gave consistently negative results when applied to sections of colonic carcinoma and previously positive primary breast tumours and lymph node secondaries. (The CEA antigen was kindly donated by Dr C. Ford and Mr J. Griffin, Surgical Immunology Unit, Clinical Oncology, Queen Elizabeth Hospital, Birmingham.)

*Assessment of sections.*—Sections stained for CEA were assessed for the number of cells showing a positive reaction; the intensity of staining was not assessed. Results were classified into 3 groups: one in which all cells were negative for CEA (− group): one in which only a small number were positive (+ group) and one in which a large number were positive (+ + group). In order to define the point of division between weakly positive (+) and strongly positive (+ +) cases in terms of the percentage of tumour cells positive for CEA and to assess how well the two groups were separated, a sample of the positive cases was formally quantitated by a cell count. The same sample was used to determine whether there was variation in CEA expression from one area to another within primary tumours and from one axillary metastasis to another within individual patients. Thus sections from 2 or 3 different areas within each of 14 primary tumours and one section from between 2 and 7 different axillary node metastases from each of 34 individual patients were assessed. All sections assessed as weakly positive (+) contained $<5\%$ CEA positive tumour cells whereas all sections considered to be strongly positive (+ +) contained $\geqslant 5\%$ positive cells. Comparison of results of CEA expression between different areas of primary tumours and between different axillary metastases from individual patients showed that in both situations there was variation in results in 18% of cases when the three categories −, + and + + were used. Most of this, however, was due to the co-existence of negative and weakly positive areas both within primary tumours and between axillary metastases. For this reason, in order to reduce the influence of inter-site variation of CEA expression to a minimum, the major comparison made in this study is based upon two groups, one in which negative (−) and weakly positive (+) cases are combined together and another which comprises only strongly positive (+ +) cases. In this way, cases in which sections contained $<5\%$ CEA positive tumour cells (including all negative cases) were compared with those in which sections contained $>5\%$ positive cells.

Sections of primary breast tumours from 290 patients (142 control and 148 treated) and axillary node secondaries from 217 patients (99 control and 118 treated) were examined for CEA and categorized into −, + or + + groups without prior reference to the clinical course of the patients.

*Other parameters.*—In addition to immunoperoxidase staining for CEA, conventionally stained sections of the primary tumours were assessed for histological grade (Bloom & Richardson, 1957). Also samples of primary tumour cytosols were analysed for oestrogen receptor content using the dextran-coated charcoal method.

*Statistical methods.*—Correlations between the CEA status and clinical course of patients were performed using life table analysis. The differences in disease free and actual survival between the various groups under study were demonstrated by using the Peto log-rank method (Peto *et al.*, 1977). For each comparison life tables were constructed and a log-rank $\chi^2$ statistic estimated. All other comparisons were performed using the conventional $\chi^2$ test. A $P$-value of $\leqslant 0{\cdot}05$ was taken to be statistically significant.

### RESULTS

### *CEA staining in primary and metastatic breast cancer*

Primary breast tumours from a total of 290 patients and axillary node secondaries from a total 217 patients were studied for CEA. Both primary and metastatic tumours were examined in 209 patients, the primary only in 81 patients and the axillary metastasis only in 8 patients. About one third of the primary tumours were strongly positive (+ +) for CEA whereas approximately one quarter of the metastic tumours were in this group (Table II). There was concordance between CEA results in primary and secondary tissue in 71% of cases.

There was no significant correlation between CEA status of either primary or secondary tissue and (i) menopausal

TABLE II.—*CEA results in primary and metastatic breast cancer*

| | CEA negative (−) | CEA positive (+) | CEA negative (−) or weakly positive (+) | Strongly CEA positive (+ +) | |
|---|---|---|---|---|---|
| Primary tumours | 111 (38) | 80 (28) | 191 (66) | 99 (34) | 290 |
| Metastatic tumours | 124 (58) | 40 (18) | 164 (76) | 53 (24) | 217 |

( ) = %.

status, (ii) size of the primary tumour, (iii) histological grade of the primary tumour or (iv) oestrogen receptor status of the primary tumour.

There was no difference morphologically between CEA positive and negative tumour cells in either primary or metastatic tissue.

*Correlation of CEA status with clinical course of patients*

(Abbreviations used: N = number in group; R = number of recurrences in group; D = number of deaths in group; − = negative for CEA; + = weakly positive for CEA; −/+ = combination of negative and weakly positive cases; + + = strongly positive for CEA.)

The median duration of follow-up of the patients in this study was 2 years 7 months (range 9 months to 4 years 7 months). At this stage there was no significant difference in recurrence free survival between the control (surgery only) and the treated (surgery + chemotherapy) groups.

*A. Primary tumours.*—Recurrence-free survival of the control group of patients in which the primary tumours were negative or weakly positive for CEA (−/+) was compared with that of control patients in which the primary tumours were strongly positive for CEA (+ +). There was no significant difference between the two groups (−/+ group, N = 92, R = 36; + + group, N = 50, R = 19; $P = 0{\cdot}80$). The group of patients that had received adjuvant chemotherapy was examined in the same way. There was again no significant difference between the two groups (−/+ group, N = 100, R = 38; + + group, N = 48, R = 15; $P = 0{\cdot}51$). The treatment and control subgroups of each category of CEA results (*i.e.* −/+, + +) were compared for recurrence-free survival. No significant differences were present in either case (−/+ group $P = 0{\cdot}39$; + + group $P = 0{\cdot}34$).

*B. Axillary node metastases.*—The same methods of analysis were applied to CEA results in axillary node metastases. Within the control group of patients recurrence-free survival was significantly better for the CEA + + group than the CEA −/+ group (Fig. 1; + + group, N = 21, R = 4; −/+ group, N = 78, R = 38; $P = 0{\cdot}036$). When the same data were analysed using actual survival there was a similar trend in favour of the group strongly positive for CEA but the difference was not significant (+ + group, N = 21, D = 1; −/+ group, N = 78, D = 12; $P = 0{\cdot}23$). When the chemotherapy group of patients was analysed for recurrence free survival, no significant difference was evident (+ +

FIG. 1.—Recurrence-free survival based on CEA results in axillary metastases. Control group only: comparison of −/+ and + + subgroups.

| | | N | R | |
|---|---|---|---|---|
| - - - | −/+ | 78 | 38 | |
| —— | + + | 21 | 4 | $P = 0{\cdot}036$. |

FIG. 2.—Recurrence free survival based on CEA results in axillary metastases. −/+ group only: comparison of control and treated subgroups.

| | N | R | |
|---|---|---|---|
| —— Control | 78 | 38 | |
| - - - Treated | 86 | 31 | $P = 0{\cdot}036$. |

group, N = 32, R = 13; $-/+$ group, N = 86, R = 31; $P = 0{\cdot}54$).

CEA $++$ and CEA $-/+$ groups were then analysed separately according to treatment group. The results of comparison of recurrence-free survival of the treated and control subgroups of patients in the CEA $-/+$ category are shown in Fig. 2. CEA $-/+$ patients in the treatment group fare significantly better than similar patients in the control group (treatment group, N = 86, R = 31; control group, N = 78, R = 38; $P = 0{\cdot}036$). A similar trend was seen for actual survival but the difference was not significant (treatment group, N = 86, D = 6; control group, N = 78, D = 12; $P = 0{\cdot}09$). When the treated and control subgroups of patients in the CEA $++$ category were compared, there was no statistically significant difference in recurrence free survival (treatment group, N = 32, R = 13; control group, N = 21, R = 4; $P = 0{\cdot}17$).

In view of the significant difference in recurrence-free survival between the treatment and control subgroups of the CEA $-/+$ category, the two components of this category, *i.e.* $-$ and $+$, were examined separately. Comparison of treatment and control groups that were negative for CEA showed no significant difference (treatment group, N = 65, R = 28; control group, N = 60, R = 28: $P = 0{\cdot}37$). However, when patients with metastases weakly positive for CEA were analysed independently according to treatment group, it was found that, despite small numbers, the recurrence-free survival was very significantly higher in the treatment group than the control group (Fig. 3; treatment group, N = 21, R = 3; control group, N = 18, R = 10; $P = 0{\cdot}005$). When the same data were analysed using actual survival there was again a significant difference (Fig. 4; treatment group, N = 21, D = 0; control group, N = 18. D = 5; $P = 0{\cdot}009$).

FIG. 3.—Recurrence free survival based on CEA results in axillary metastases. + group only: comparison of control and treated subgroups.

| | N | R | |
|---|---|---|---|
| —— Control | 18 | 10 | |
| – – – Treated | 21 | 3 | $P = 0{\cdot}005$. |

FIG. 4.—Actual survival based on CEA results in axillary metastases. + group only: comparison of control and treated subgroups.

| | N | D | |
|---|---|---|---|
| —— Control | 18 | 5 | |
| – – – Treated | 21 | 0 | $P = 0{\cdot}009$. |

In order to exclude the possibility of bias due to unequal distribution of known prognostic factors, groups showing significant differences were examined for the following: menopausal status, size of primary tumour, histological grade and oestrogen receptor status. These factors were found to be evenly distributed between the groups under comparison. Finally, the proportion of recurrences in each group that occurred at locoregional or distant sites was compared. In every group between 60 and 75% of the recurrences were locoregional, between 25 and 36% were distant and in 0–10% both sites were involved.

### DISCUSSION

The potential benefit of adjuvant chemotherapy has to be set against the cost of acute toxicity (Palmer *et al.*, 1980), the possibility of long-term organ damage and induction of second tumours (Reimer *et al.*, 1977; Lerner, 1978; Valagussa *et al.*, 1980) and economic factors. On the basis of present evidence it seems unlikely that adjuvant chemotherapy will be of equal benefit to all patients presenting with breast cancer. It is clear, therefore, that such treatment is only justifiable if it substantially improves prognosis and that there is a need for accurate discrimination between those patients who will and those who will not benefit from chemotherapy. A number of important prognostic factors have been defined for operable breast cancer but their value as predictors of response to chemotherapy remains to be elucidated. The therapeutic value of the chemotherapy regimen used in the West Midlands Oncology Association Trial of Adjuvant Chemotherapy for Operable Breast Cancer will only become clear when further follow-up allows analysis of the 5- and 10-year survival figures. However, interim analyses may reveal certain parameters which might be of potential value in the selection of patients for chemotherapy.

This study was designed to determine whether the production of CEA by primary tumours and their nodal metastases could be used in this way. From the results presented, it is evident that the presence of CEA in primary tumours is of no detectable prognostic value. However, the CEA status of the axillary metastases does seem to have some bearing upon the clinical course of patients. Those in the control group were shown to have a significantly better chance of remaining free of recurrence if their nodal metastases were strongly positive for CEA ($>5\%$ positive tumour cells), than if they were in the combined negative and weakly positive group (*i.e.* $<5\%$ positive cells). In addition, no further advantage in recurrence-free survival was produced by adjuvant chemotherapy in the group with nodal metastases strongly positive for CEA. It would be interesting to extend the analysis by subdividing the strongly positive category using for example 25% CEA positive cells as the cut-off point. At present however there is an insufficient number of patients in this category to permit satisfactory statistical analysis of such subgroups.

In contrast, chemotherapy did result in significantly better recurrence free survival when given to patients whose nodal secondaries contained $<5\%$ CEA-positive cells (*i.e.* the $-/+$ group) compared with the control group. Also when those patients with weakly positive results were examined in isolation, adjuvant chemotherapy was shown to produce a highly significant improvement in both recurrence-free survival and actual survival in comparison with the control group. However, no significant improvement in the prognosis of patients with CEA-negative nodal metastases was seen during this period of follow-up. From an assessment of inter-site variation in CEA expression (described under "assessment of sections") it is apparent that the negative and weakly positive groups are not entirely homogeneous. In some cases classed as having negative axillary metastases, more extensive sampling of nodes may have revealed other metastases that were weakly positive for CEA. Nevertheless these 2 categories of CEA results ($-$ and $+$) in the axillary metastases, based on limited sampling (1–3 involved nodes), do seem to define groups of patients that behave differently in terms of their response to chemotherapy.

None of the significant differences discussed above could be explained by unequal representation of certain good prognostic features within the groups compared, including small size of primary tumour, good histological grade and the presence of oestrogen receptor in the primary tumour. Furthermore, since it has been shown that survival after local recurrence is longer than survival after distant metastasis (Karabali-Dalamaga *et*

*al.*, 1978), the proportion of recurrences at each of these sites was determined in each of the groups. It was found, that in every group approximately two-thirds of recurrences were locoregional and one-third occurred at distant sites. Therefore, there is no reason to suspect that differences in mean duration of survival were produced by mal-distribution of any of these prognostic factors.

A possible explanation for the observed relationship between axillary node CEA status and prognosis and response to chemotherapy is suggested by data from *in vitro* studies (Drewinko & Yang, 1976, 1980; Ellison *et al.*, 1977; Rutzky *et al.*, 1979). It has been shown that rapidly dividing tumour cells fail to produce CEA whereas cells in the stationary phase of growth are capable of CEA production. It could be argued, that axillary metastases which contain large numbers (*i.e.* $> 5\%$) of CEA-producing cells are associated with a favourable prognosis because a significant proportion of the metastatic cells are quiescent, the growth fraction is small and the growth rate of metastases is slow. This could explain the failure of chemotherapy to increase recurrence-free survival in this group, since most cytotoxic drugs have greater toxicity for rapidly proliferating cells (Madoc-Jones & Bruce, 1967; Goldenberg *et al.*, 1971; Barranco & Novak, 1974; Twentyman & Bleehen, 1975). Conversely, those patients with metastases that contain only a small proportion of CEA positive cells or none at all might be expected to have a worse prognosis because of the more aggressive nature of the tumour, and would be expected to show a good response to chemotherapy because of the larger proportion of dividing cells. Although, this proved true for patients with metastases that were weakly positive for CEA, there was no improvement in recurrence-free survival in CEA-negative cases. One possible interpretation is that the CEA-negative cases represent the most aggressive end of the spectrum of tumour growth and that chemotherapy was not capable of controlling the disease.

An alternative explanation of the findings is that CEA may induce an immune response and that this could affect the rate of progression of disease. However, the evidence for this is somewhat tenuous (Carrel *et al.*, 1977; Hammarstrom *et al.*, 1977; Kapsopoulou-Dominos & Anderer, 1979; Staab *et al.*, 1980*b*).

Whatever the explanation of these results, it does appear that the CEA status of axillary node metastases from patients with node-positive operable breast cancer may be of some clinical relevance in relation to prognosis and selection of patients for chemotherapy. The immunoperoxidase technique used throughout this study could be performed by any routine pathology laboratory and is relatively inexpensive. Clearly, it is important to repeat these analyses periodically as the duration of follow-up increases in order to determine whether the differences described above persist, and to detect any further trends that are not yet apparent which might be of clinical value.

We thank all of the surgeons who have entered patients into the study. They are Mr J. M. Morrison, Mr G. D. Oates, Mr S. Glick, Mr H. Kramer, Mr J. D. Hennessy, Mr R. S. Rihan, Mr W. M. Lien, Mr D. R. Thomas, Mr T. A. Waterworth, Mr H. C. de Castella, Mr K. D. Fortes-Mayer, Mr P. Armitstead, Mr N. J. Dorricott, Mr R. M. Baddeley, Mr M. R. B. Keighley, Professor P. G. Bevan, Mr I. A. Donovan, Mr J. G. Temple, Mr E. R. Monypenny, Mr L. J. Lawson, Mr J. H. Burman, Mr R. E. Gibbins, Mr R. T. Marcus, Mr J. D. Marsh, Mr M. D. Lord, Mr J. Alexander-Williams, Mr G. A. Court, Mr M. L. Obeid, Mr J. R. Moffatt, Mr E. W. Gillison, Mr G. F. Grave, Mr R. W. Tudor, Mr R. W. Parker, Mr H. T. Williams, Mr A. R. Leask, Mr F. R. Hurford and Mr M. E. Winstone. In addition we are grateful to the pathologists at the various contributing centres, to Dr R. A. Walker for coordinating the collection of the histological material, grading the primary tumours and generous technical advice, to Dr D. Cove and Dr A. Hughes for supervising the oestrogen receptor analyses and to the numerous other staff who have contributed to the running of the trial. Our thanks are also due to Miss Jill Spencer for typing the manuscript.

S.R.S. was supported initially by a Sheldon Clinical Research Fellowship and subsequently by a MRC Clinical Training Fellowship.

### REFERENCES

Barranco, S. C. & Novak, J. K. (1974) Survival responses of dividing and non-dividing mammalian cells after treatment with hydroxyurea, arabinosylcytosine or adriamycin. *Cancer Res.*, **34**, 1616.

BIVINS, B. A., MEEKER, W. R. & GRIFFIN, W. O. (1975) Carcinoembryonic antigen (CEA) levels and tumour histology in colon cancer. *J. Surg. Res.*, **18**, 257.

BLOOM, H. J. G. & RICHARDSON, W. W. (1957) Histological grading and prognosis in breast cancer. *Br. J. Cancer*, **11**, 359.

CARREL, S., DELISLE, M-C. & MACH, J-P. (1977) Antibody-dependent cell-mediated cytolysis of human colon carcinoma cells induced by specific antisera against carcinoembryonic antigen. *Cancer Res.*, **37**, 2644.

DREWINKO, B. & YANG, L. Y. (1976) Restriction of CEA synthesis to the stationary phase of growth of cultured human colon carcinoma cells. *Exp. Cell Res.*, **101**, 414.

DREWINKO, B. & YANG, L. Y. (1980) Observations on the synthesis of carcinoembryonic antigen by an established human colonic carcinoma cell line. *Oncology*, **37**, 336.

ELLISON, M. L., LAMB, D., RIVETT, J. & NEVILLE, A. M. (1977) Quantitative aspects of carcinoembryonic antigen output by a human lung carcinoma cell line. *J. Natl Cancer Inst.*, **59**, 309.

FALKSON, H. C., VAN DER WATT, J. J., PORTUGAL, M. A., PITOUT, M. J. & FALKSON, G. (1978) Carcinoembryonic antigen in patients with breast cancer. An adjunctive tool to monitor response and therapy. *Cancer*, **42**, 1308.

FALKSON, H. C., VAN DER WATT, J. J., PORTUGAL, M. A., SCHOEMAN, H. S. & FALKSON, G. (1979) Role of plasma carcinoembryonic antigen in evaluating patients with breast cancer treated with adjuvant chemotherapy. *Cancer Treat. Rep.*, **63**, 1303.

GOLDENBERG, G. J., LYONS, R. M., LEPP, J. A. & VANSTONE, C. L. (1971) Sensitivity to nitrogen mustard as a function of transport activity and proliferative rate in L5178Y lymphoblasts. *Cancer Res.*, **31**, 1616.

HAMMARSTROM, S., TROYE, M., WAHLUND, G., SVENBERG, T. & PERLMANN, P. (1977) K cell mediated lysis of cultured colon carcinoma and urinary bladder carcinoma cells induced by monospecific antisera against carcinoembryonic antigen (CEA) and two CEA-related normal glycoproteins. *Int. J. Cancer*, **19**, 756.

KAPSOPOULOU-DOMINOS, K. & ANDERER, F. A. (1979) Circulating carcinoembryonic antigen immune complexes in sera of patients with carcinomata of the gastrointestinal tract. *Clin. Exp. Immunol.*, **35**, 190.

KARABALI-DALAMAGA, S., SOUHAMI, R. L., O'HIGGINS, N. J., SOUMILAS, A. & CLARK, C. G. (1978) Natural history and prognosis of recurrent breast cancer. *Br. Med. J.*, **ii**, 730.

LAMERZ, R., LEONHARDT, A., EHRHART, H. & LIEVEN, H. V. (1980) Serial carcinoembryonic antigen (CEA) determinations in the management of metastatic breast cancer. *Oncodev. Biol. Med.*, **1**, 123.

LERNER, H. J. (1978) Acute myelogenous leukaemia in patients receiving chlorambucil as long-term adjuvant chemotherapy for stage 11 breast cancer. *Cancer Treat. Rep.*, **62**, 1135.

MADOC-JONES, H. & BRUCE, W. R. (1967) Sensitivity of L cells in exponential and stationary phase to 5 fluoro-uracil. *Nature*, **215**, 302.

MORRISON, J. M., HOWELL, A., GRIEVE, R. J., MONYPENNY, I. J., MINAWA, A. & WATERHOUSE, J. A. (1981) The West Midlands Oncology Association Trials of Adjuvant Chemotherapy for Operable Breast Cancer. In *Adjuvant Therapy of Cancer III* (Eds Salmon & Jones). New York: Grune & Stratton. p. 403.

O'BRIEN, M. J., BRONSTEIN, B., ZAMCHECK, N., SARAVIS, C. BURKE, B. & GOTTLIEB, L. S. (1980) Cholestasis and hepatic metastases: a factor contributing to extreme elevations of carcinoembryonic antigen. *J. Natl Cancer Inst.*, **64**, 1291.

PALMER, B. V., WALSH, G. A., MCKINNA, J. A. & GREENING, W. P. (1980) Adjuvant chemotherapy for breast cancer: side effects and quality of life. *Br. Med. J.*, **281**, 1594.

PETO, R., PIKE, M. C., ARMITAGE, P. & 7 others (1977) Design and analysis of randomized clinical trials requiring prolonged observation of each patient. *Br .J. Cancer*, **35**, 1.

REIMER, R. R., HOOVER, R., FRAUMENI, J. F. & YOUNG, R. C. (1977) Acute leukaemia after alkylating-agent therapy of ovarian cancer. *N. Engl. J. Med.*, **297**, 177.

RUTZKY, L. P., TOMITA, J. T., CALENOFF, M. A. & KAHAN, B. D. (1979) Human colon adenocarcinoma cells. III. *In vitro* organoid expression and carcinoembryonic antigen kinetics in hollow fiber culture. *J. Natl Cancer Inst.*, **63**, 893.

SHOUSHA, S. & LYSSIOTIS, T. (1978) Correlation of carcinoembryonic antigen in tissue sections with spread of mammary carcinoma. *Histopathology*, **2**, 433.

SHOUSHA, S., LYSSIOTIS, T., GODFREY, V. M. & SCHEUER, P. J. (1979) Carcinoembryonic antigen in breast cancer tissue: a useful prognostic indicator. *Br. Med. J.*, **i**, 777.

STAAB, H. J., AHLEMANN, L. M., KOCK, H. L. & ANDERER, F. A. (1980*a*) Serial carcinoembryonic antigen (CEA) determinations in the management of patients with breast cancer. *Oncodeve. Biol. Med.*, **1**, 151.

STAAB, H. J., ANDERER, F. A., STUMPF, E. & FISCHER, R. (1980*b*) Are circulating CEA immune complexes a prognostic marker in patients with carcinoma of the gastrointestinal tract? *Br. J. Cancer*, **42**, 26.

STEWARD, A. M., NIXON, D., ZAMCHECK, N. & AISENBERG, A. (1974) Carcinoembryonic antigen in breast cancer patients: serum levels and disease progress. *Cancer*, **33**, 1246.

TORMEY, D. C., WAALKES, T. P., SNYDER, J. J. & SIMON, R. M. (1977) Biological markers in breast carcinoma. III. Clinical correlations with carcinoembryonic antigen. *Cancer*, **39**, 2397.

TWENTYMAN, P. R. & BLEEHEN, N. M. (1975) Changes in sensitivity to cytotoxic agents occurring during the life history of monolayer cultures of a mouse tumour cell line. *Br. J. Cancer*, **31**, 417.

VALAGUSSA, P., SANTORO, A., KENDA, R. & 5 others (1980) Second malignancies in Hodgkin's disease: a complication of certain forms of treatment. *Br. Med. J.*, **280**, 216.

WALKER, R. A. (1980) Demonstration of carcinoembryonic antigen in human breast carcinomas by the immunoperoxidase technique. *J. Clin. Pathol.*, **33**, 356.

ZAMCHECK, N., DOOS, W. G., PRUDENTE, R., LURIE, B. B. & GOTTLIEB, L. S. (1975) Prognostic factors in colon carcinoma. Correlation of serum carcinoembryonic antigen level and tumour histopathology. *Hum. Pathol.*, **6**, 31.